# The Immediate Treatment of the Uncovered Pulp

**Published:** 1895-04

**Authors:** Louis Jack

**Affiliations:** Philadelphia.


THE
AMERICAN JOURNAL
—OF—
DENTAL SCIENCE.

VOL. XXVIII. BALTIMORE, APRIL, 1895. NO. 12.

ARTICLE I.

### THE IMMEDIATE TREATMENT OF THE UNCOVERED PULP.

BY PROFESSOR LOUIS JACK, PHILADELPHIA.

When accidental exposure has been made, tincture of calendula, one part to four of water, should be applied as soon as bleeding has ceased. The pulp should then be immediately dressed and capped in the manner to be described later. If only a slight injury has been inflicted, the cavity may be filled at once with metal, having regard to the strength, the placement, and the fixation of the cap used to defend from compression, as will later be described. Here the fixation of the cap is best made by covering it with a broad mat of gold-foil; after adapting this to the margins one may proceed to complete the filling. In respect to this class of exposures there should be no concern as to the success of the treatment, providing the pulp was in a healthy condition at the time of the accident.

When the pulp has been fully recovered, the cavity should be washed clean with tepid water, scarcely protected from the fluids of the mouth with rubber-dam, dried and lightly glled with a pledget of lint saturated with a mild disinfectant. From your knowledge of the causes of caries and of the concomitant invasion of the zone of dentine immediately beneath the caries by bacteria and micrococci, you will recognize that some means of sterilization must be resorted to. It is necessary in the treatment of ordinary cavities, hence you will perceive how much more it is here required. On account of the impatience of the pulp to medication, we have to be very careful in the selection of the sterilizing agent. There is none with which I am acquainted that is less irritating than hydronaphthol. This should not be stronger than 1 to 300 parts of water. The saturated pledget of cotton may remain in the cavity during the procedures of the preparation of the dressing paste, the selection of the cap, etc.

When these preparations are complete the cavity should be again dried, the drying being finished by a few puffs of warmed air. The point of exposure and the adjacent dentine is now touched with a tent of lint, filled with carbolic acid and oil of cloves, aa. The effect of this is to coagulate to a superficial degree the point of exposure. This practice is largely empirical. It may be avoided where no disturbance has previously existed; but where there are evidences of irritation, it has appeared by my experience to be indispensable.

The first impression made on the minds of those who have been taught that carbolic acid is an irritant to pulp-tissue, is that this proposition would appear to be questionable. On this point I have had much experimental knowledge. In the treatment of earlier cases, under the prejudice that carbolic acid was irritating, I employed a weak solution of this agent. The results were not favorable, for the reason that in the dilute form it did not coagulate the surface of the exposed point, and was therefore liable to be absorbed, becoming consequently irritating to the pulp.

Pure, or nearly pure, carbolic acid combined with oil of cloves, as here recommended, at once produces slight coagulation of the surface of the organ, and appears from the absence of irritation to enter no further into the tissue. You will not fail to observe the sterilizing and anesthetic value of this combination.

In respect of the sterilization of the dentine, questions may arise whether this may be sufficient to cover the necessities of the case in this respect. First, whether complete sterilization is thus effected; second, whether the pulp may not already have become so far inoculated by bacteria as to render its normal condition an impossibility; and whether, under these circumstances, attempts of superficial sterilization of the dentine can be of any service.

You will not fail to keep in view that the treatment is confined to cases in which it is evident the pulp-tissue is probably not under much irritation. It is hyperemic, and consequently in a hyperesthetic state congestion has not occurred, nor does inflammation exist. Therefore, the inference is reasonably certain that after the soft caries is removed we may safely sterilize the surface of the dentine, and leave the vital force of the pulp to take care of whatever slight bacterial invasion may have reached that tissue. I assume here that your instruction has included the observed fact that healthy tissues have the power of mastering the invasion of non-pathogenic bacteria.

### THE CAP.

A prominent feature in conservative treatment of the pulp is the means to protect it from pressure, and because of this necessity the means mostly used have given this mode of treatment the appellation of "capping the pulp." The object of the cap is the avoidance of compression. Various means have been used to accomplish this. A common method has been to cover by a dressing the point of exposure; the bottom of the cavity being then lined with oxichlorid of zinc or phosphate of zinc, a coating of guttapercha varnish being interposed between the dressing and the protective material. As this means is complicated, somewhat uncertain, not applicable in shallow cases, and in those very difficult of access, I have preferred to depend on a metal cap as being simpler and better under control.

This kind of cap not only insures to prevent compression, but serves to contain the dressing, which completely occludes the space between the cap and the pulp. These caps may be made of tin platina or of gold, the former being preferable. In form they should be circular to meet the generality of cases, and oval to accommodate broad exposures. They are most easily made by hunching them out with the round and oval leather punches of the hardware shops on the end of a block of hard wood. This action gives them sufficient concavity for the purpose. For ordinary purposes they should be quite thin, but when it is considered safe to use gold fillings the cap should be sufficiently thick to resist the force of the packing.

### The Dressing.

It is necessary that the space under the cap should be completely closed, otherwise it would fill with fluid effused from the pulp, which quickly would undergo putrefactive changes resulting in the evolution of gas and consequently of compression of the organ beneath. In the earliest attempts at capping the pulp this became the condition, the only thought then being to protect from the pressure of the instruments. It was then generally supposed that the pulp underwent projection into the space and became strangulated, but all the indications now point to compression by gases being the cause of the disturbance.

The dressing I have found most acceptable is formed by rubbing up oxid of zinc into a paste with carbolic acid and oil of cloves of equal measure. The consistency of this should be such that, while being so plastic that the paste will flow out around the cap when it is gently pressed in position, it will not be so thin as to flow out of the cap when it is held on its edge.

The ingrediency of the dressing is based on these considerations. The menstruum is antiseptic, and possesses some anesthetic value. It also remains unchanged within the space, and in time becomes, from the dissipation of the menstruum, somewhat firm in its character. Therapeutic action of the menstruum is mild, and is employed because it is slowly given up by the oxid of zinc, and, therefore, makes an acceptable dressing.

I would call your attention to the value of the combination of hypophosphate of lime with zinc oxid, aa, with the previously stated menstruum. This I have used in many of my earlier experiments, and have lately returned to it in less promising character than those considered within the lines of easy treatment.

### PLACING THE CAP IN POSITION.

Placing the cap in position is a step in the treatment requiring care. It should be assured that it is of sufficient size to pass well beyond the borders of the exposed organ, and in the proximate cavities it should cover the pulp-wall of the cavity without intruding on the marginal walls. If there is a single exposure it should be round; if two corone are exposed, either two caps should be laid or one oval employed. In molars, usually where two points are exposed, two caps are generally best; in the bicuspid one oval under the same circumstances. The cap should be inserted edgewise in such manner that as it is laid in place the excess of dressing may flow out at the margin toward the operator. This is to prevent excessive pressure, and to prevent air being included beneath the dressing, which would prevent complete apposition of the dressing with the pulp.

If there is easy access the cap may be laid in place with fine-pointed pliers--notably the Bogue plier; but, generally, it is preferable to previously coat the convex side of the metal with yellow wax, when, with an instrument adapted to the case it may be carried into position, and then placed in the manner described. It should next be pressed into position with sufficient force to bring the margins in contact with the dentine. Any excess of dressing should be taken away by light touches of an excavator, and when the cavity is to be filled temporarily it is better to fix the cap in place by flowing over it a little chloro-percha, which, when dried, prevents disturbance of its position in the filling procedure.

You should be careful that when the pulp is found exposed in a depression, as occurs sometimes in the molars, this depression should be filled nearly or quite to a level with the floor of the cavity by taking a little of the dressing on a suitable instrument and carefully filling this point; otherwise, when the cap is placed, the paste may not find its way into contact with the pulp. In a few instances, in my earlier experience, I have been obliged to go over the case and recap on account of this difficulty.

At the moment of placing the cap, as the paste is yielding under the gentle pressure of forcing the edges of the cap in contact with the dentine, a little pain will be observed; but unless the paste is too stiff, no compression of the pulp will be caused.

### FILLING THE CAVITY.

Whether the cavity be filled temporarily or permanently depends on the prognosis. This depends on the systemic conditions and the state of the pulp at the time of treatment.

To those of small experience in this line of treatment it would not be safe to attempt permanently stopping the cavity, except in accidental exposures, and in cases where no previous disturbance can be elicited. Even in the latter class it is generally best to delay permanent closure by a conductor of heat till after an experience of a year or more with a non-conducting stopping. At the end of this time the filling may be nearly all removed, care being taken not to disturb the capping, when, with suitable precaution, a metallic filling may be inserted.

I have many capped pulps beneath gold fillings, where they have remained, without the occurrence of any irritation, for many years. But the utmost caution must be used in making the selection of cases to be filled permanently at the time of the capping of the pulp.

Generally it is safest to fill the cervical part with gutta-percha stopping, carrying the material over the cap, and then to complete the filling with zinc phosphate. In this way, with an occasional renewal of this temporary work, cases may be carried forward from ten to fifteen years.

They may, however, be closed permanently and safely after an experimental trial of five years where no irritation has appeared.

This brings us to consider the conditions which take place with the pulp after the procedures which I have described. Concerning this there is no certain data.

Obviously the most desirable result would be the conversion of the exposed surface of the pulp into secondary dentine. This result I have known to take place within two years, and I have opened for examination cases which have been capped, without the occurrence of any disturbance beyond the occasional impressibility by cold for periods as long as fifteen years, where no such change had taken place.

That in many cases recovery is by secondary deposits my records present a considerable number of instances. It is not remarkable, however, that the pulps may remain in a state of quiescence for years, when it is considered that in slowly advancing caries the pulp will often be exposed for long periods without the occurrence of any sign of irritation, unless, by the portion of the mouth of the cavity, the pulp has been subjected to the pressure of food.

It may be concluded that, whether the pulp becomes protected by secondary deposits or acquires complete quiescence, conservative treatment has considerable advantage over immediate devitalization. Still, in this connection, I must repeat the necessity for the careful selection of subjects to be treated, and also for proper regard to the apparent condition of the pulp itself.

### AFTER TREATMENT.

It is presumed that the judicious practitioner has made careful selection of the cases to be treated conservatively, and that he will early decide on the evident condition of those which preclude recovery. Some of the most promising cases will not yield to treatment. This consideration requires close observation of the cases for some time after treatment. The patient should be instructed to return for consultation should reflected pain occur or should the teeth become impatient of cold applications. If this should be apparent, it is a sign of needed care to avert increased disturbance.

A most marked form of reflected pain is felt in the ear, and this frequently appears before the temperature sense has become aggravated. So much importance should be attached to this symptom of pulp disturbance that the first question to be asked a patient appearing with pain, or on approaching a suspected pulp, is: Have you had any pain in the ear of that side? Reflection to the ear occurs often long in advance of similar pain in other branches of the third pair.

Where the teeth has been impressed by cold, either before the treatment or afterward, an application should be made to the gum over the tooth, of tincture of aconit, two parts; chloroform, one part. The mode of application is important. A pledget of cotton or muslin to cover an area of one-half by three fourths of an inch should be saturated, then squeezed out nearly to dryness between folds of a napkin to prevent an excess flowing over the mouth and with the saliva entering the fauces, to which it is extremely irritating as well as unnecessarily medicating the patient. Before the pledget is applied the surface of the gum should be cleansed of the coat of mucus covering it, otherwise the remedy will fail to come in contact with the membrane. It is equally important that dryness of the surface be secured. This application should be maintained for from twelve to fifteen seconds. If allowed to remain too long on the part ulceration is established. The general after-treatment consists in the repeated application of aconit, the application not being made at the same point more frequently than after intervals of forty-eight hours. When it is desired to increase the counter-irritation the gum may be scarified very superficially by quick, light movement of a small scalpel. The patient should be instructed to avoid subjecting the tooth to extremes of temperature in either direction. The control period of conservatively-treated cases is within the first fortnight after the capping.

I have, however, had them appear after several years of complete quiescence with initial disturbances, and then yield to treatment. It sometimes becomes necessary to open the cases and recap. This usually occurs when in reviewing the case it is considered that some oversight has occurred. There may have been two exposures. The cap may not have completely covered the exposed part. There may have become compression from forcing the cap. It may have been displaced, the case may be determined to go down the gamut of irritation, and in despair we sterilize again and make another trial.

The most careful records of cases should be kept with a relation of the condition and of the controlling symptoms in a book kept for this purpose. Should subsequent irritation occur a new diagnosis may be formed from the recorded facts and the new conditions. I am careful to have this class of cases carried forward to the examination chart at each recurring periodic examination of the teeth. They are marked in symbol with red ink to prevent the unnecessary removal of temporary fillings, to explain the reason for their presence, and thus to avoid the accident of an unnecessary uncovering of the pulp in such cases.—*International.*

